# Antimicrobial susceptibility pattern of *Shigella* spp. isolated from children under 5 years of age attending tertiary care hospitals, Nepal along with first finding of ESBL-production

**DOI:** 10.1186/s13104-017-2512-1

**Published:** 2017-06-05

**Authors:** Subhash Dhital, Jeevan Bahadur Sherchand, Bharat Mani Pokharel, Keshab Parajuli, Shyam Kumar Mishra, Sangita Sharma, Hari Prasad Kattel, Sundar Khadka, Sulochana Khatiwada, Basista Rijal

**Affiliations:** 1 National Public Health Laboratory, HIV Reference Unit, Kathmandu, Nepal; 2 Department of Microbiology, Tribhuvan University Teaching Hospital, Kathmandu, Nepal; 3 Department of Microbiology, Universal Medical College, Bhairahawa, Nepal

**Keywords:** *Shigella*, Antibiotic resistance, ESBL, Nepal

## Abstract

**Background:**

*Shigella* is an important cause of bacterial gastroenteritis in resource-poor countries. The treatment of shigellosis mostly requires antibiotics. However, the increase of multidrug resistance along with emergence of extended-spectrum β-lactamase and ciprofloxacin resistance among *Shigella* spp. has challenged the situation. This study was conducted to determine the distribution of species and antibiotic susceptibility pattern of *Shigella* species isolated from stool specimen among children less than 5 years of age in Nepal.

**Results:**

Out of total 717 stool samples collected, 15 cases of *Shigella* spp. was isolated which includes 12 *S. flexneri* and 3 *S. sonnei*. Multidrug resistance was found among 13(86%) of the isolates. One of the isolates of *S. flexneri* was found to be ESBL-producer with MIC >256 mg/L for cefixime.

**Conclusion:**

The high occurrence of multidrug resistance among *Shigella* spp. along with a case of ESBL-production for the first time in Nepal alarms the concerns about dissemination of the resistant isolates. So, systemic monitoring of the antimicrobial susceptibility pattern of *Shigella* spp. is becoming crucial to guide therapy.

## Background

*Shigella* is an important cause of bacterial gastroenteritis in resource-poor countries where children less than 5 years of age are major affected population [1]. Antimicrobial agents can lessen the severity of disease and potentially prevent lethal complications; however, sulphonamide–trimethoprim, tetracycline, ampicillin are no longer recommended for empirical treatment because of the high chances of resistance [2]. In addition, the increase of multidrug resistance along with emergence of extended-spectrum β-lactamase (ESBL) and/or ciprofloxacin resistance among *Shigella* spp. has made the situation challenging [3–6].

In current scenario, majority of the members of the family *Enterobacteriaceae* were shown to produce ESBL including *Shigella*. Third-generation cephalosporin-resistant *S. flexneri* isolate was first reported from a stool sample of a 16-month old child in Paris in 1995 [7]. In the recent years, various ESBL-producing *Shigella* were also reported from Korea [8], Argentina [9], Vietnam [10], Turkey [11] and India [12].

The free access of antibiotics from pharmacy without prescription, lack of antibiotic policy and dedicated laboratory for susceptibility testing and surveillance is leading to distressing public health threat of antibiotic resistance in Nepal. The prevalence of ESBL in Nepal is found to be 13–24% in different clinical isolates, predominantly *Escherichia coli* [13–15]. ESBLs are beta-lactamases capable of conferring bacterial resistance to the penicillins, first-, second-, and third-generation cephalosporins, and aztreonam except the cephamycins or carbapenems with various genotypes such as CTX-M, TEM, SHV, OXA [16]. Though extended-spectrum β-lactamase production in *Shigella* has not been reported from Nepal, the transfer of ESBL genes to *Shigella* spp. is possible through mobile genetic elements and plasmids [17].

This study was conducted to determine the distribution of species and antibiotic susceptibility pattern of *Shigella* species isolated from stool specimen of children less than 5 years of age in Nepal.

## Methods

**Fig. 1:**
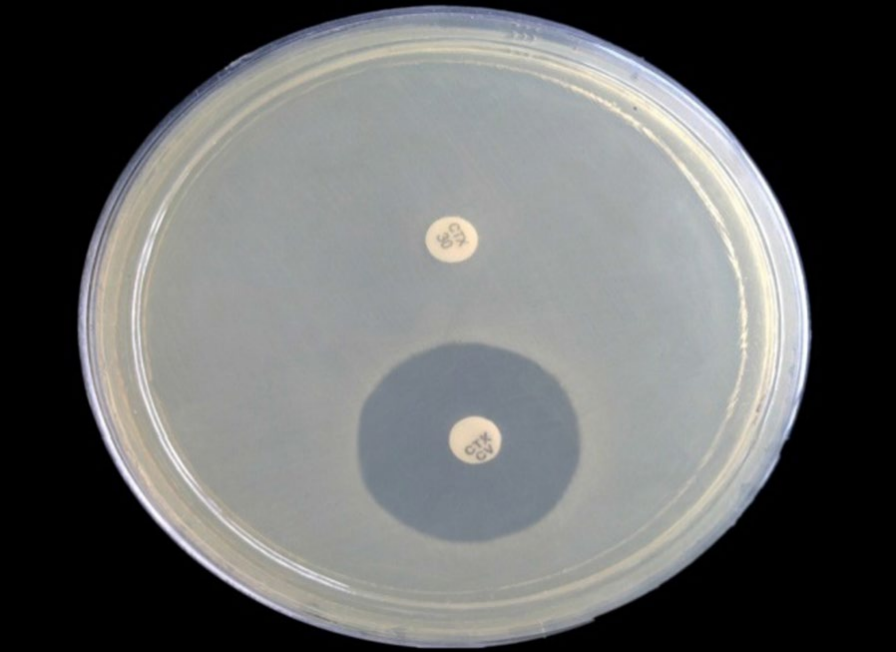
Extended spectrum β-lactamase (ESBL) detection by combination disk method

Single stool samples were collected from the children less than 5 years of age attending two referral hospitals in Kathmandu, Tribhuvan University Teaching Hospital (TUTH) and Kanti Childrens’ Hospital, from January to December 2014, with acute diarrheal illness. These hospitals are large centers in the country taking care of around 1500 acute diarrheal cases in observation ward and OPD services in a year. In the study, children admitted in hospital for longer than 3 days and/or in antibiotic use were followed as exclusion criteria. The laboratory investigation was performed following the technical guidelines of American Society for Microbiology at Department of Microbiology, TUTH [18]. The direct inoculation of stool sample was done in Xylose Lysine Deoxycholate Agar (XLD) while 6 h enriched Gram Negative broth were cultured into MacConkey agar (MA) and incubated at 37 °C for 24 h. Non lactose fermenting pale colonies from MA or pink colonies from XLD were tested for indole production, citrate utilization, urease production, lysine decarboxylation, acid and gas production with sugar utilization and motility. Presumptive *Shigella* isolates were serotyped based on serological slide agglutination, using the polyvalent antisera A–D (*Shigella* Antisera, Denka Seiken, Japan). The antibiotic sensitivity tests of *Shigella* spp. were performed using following antibiotic disks (Hi-media, Mumbai, India): amoxicillin (10 μg), nalidixic acid (10 μg), cotrimoxazole (1.25 μg/23.75 μg), ciprofloxacin (5 μg), azithromycin [19], tetracycline (10 μg), cefixime (5 μg), ceftriaxone (30 μg), ceftazidime (30 μg), ampicillin/sulbactam (10 μg/10 μg) and interpreted as recommended by CLSI [20]. The isolates having resistant zone diameters against cefixime, ceftriaxone or ceftazidime were further tested for Minimum Inhibitory Concentration (MIC) of cefixime by agar dilution and ESBL was confirmed by phenotypic disk diffusion methods, double disk synergy test and combination disk method, depicted in Fig. 1 as described by CLSI [20]. For combination disk method, ≥5 mm increase in a zone diameter for either third generation cephalsporin tested in combination with clavulanic acid versus its zone when tested alone was used as interpretative criteria to confirm an ESBL-production.

## Results

In the study, total fifteen *Shigella* isolates were identified (2.1% of total cases). *Shigella flexneri* was isolated from 12 cases whereas *Shigella sonnei* was isolated from three cases.

The antibiotic sensitivity pattern of *Shigella* spp. depicts that 33% of the isolates were sensitive to amoxicillin, cotrimoxazole and ciprofloxacin and one isolate is resistant to third generation cephalosporin, which is presented in Table 1.

**Table 1:** Antimicrobial susceptibility pattern of *Shigella*

Antibiotics	*Shigella flexneri* (n = 12)		*Shigella sonnei* (n = 3)	
	Sensitive	Resistant	Sensitive	Resistant
Amoxycillin	2	10	3	0
Nalidixic acid	3	9	0	3
Cotrimoxazole	5	7	0	3
Ciprofloxacin	5	7	0	3
Tetracycline	4	8	2	1
Azithromycin	8	4	3	0
Cefixime	11	1	3	0
Ceftazidime	11	1	3	0
Ceftriaxone	11	1	3	0
Ampicillin sulbactam	4	8	3	0

### MDR pattern of S*higella* spp.

Out of total 15 isolates, 13 isolates were found to be multidrug resistant representing 86% of the total isolates. The MDR pattern of *Shigella* spp. is presented in Table 2.

**Table 2:** MDR pattern of *Shigella* spp.

MDR pattern	Number of isolates
Amoxycillin + nalidixic acid + cotrimoxazole	3
Amoxycillin + nalidixic acid + ciprofloxacin	5
Amoxycillin + cotrimoxazole + ciprofloxacin	3
Nalidixic acid + cotrimoxazole + ciprofloxacin	7

### MIC of cefixime

Out of 15 isolates, 14 isolates had cefixime MIC ≤0.5 mg/L and one of the isolate had >256 mg/L.

### ESBL-production

One of the *S. flexneri* isolate was found to be ESBL-producer, in fact a multidrug resistant, being resistant to ciprofloxacin, cotrimoxazole and third generation cephalosporin.

## Discussion

The study was conducted from January to December 2014 among children less than 5 years suffering from acute diarrhea, attending inpatient and outpatient department of Tribhuvan University Teaching Hospital and Kanti Children’s Hospital. In the study, *Shigella* spp. were isolated from 2.1% of total cases*. Shigella flexneri* was isolated from 12 cases whereas *S. sonnei* was isolated from three cases. Similar to this study, Shah et al. reported that *Shigella* species accounted for 2.24% of total cases and *S. flexneri* was the only isolated species [21]. In accordance to the present study, *S. flexneri* has been found to be major cause of shigellosis in developing countries [1]. Zhang et al. reported *S. flexneri* among 78.7% cases from China [6]. However, in a study conducted at Eastern Nepal by Bhattacharya et al. [22] the predominant isolate was *Shigella dysenteriae* (73.7%), followed by *S. flexneri* (23%) and *Shigella boydii* (4%).

In studies made in Nepal, from 1999 to 2002 *S. flexneri* accounted for 38% and was behind *S. dysenteriae* (42%) while in study made from 2003 to 2005, *S. flexneri* (43.22%) was predominant with *S. dysenteriae* occupying 41.52%. Indeed, authors have marked the shift of predominant species to *S. flexneri* by 2005 [23].

Similar type of serogroup changes have been reported in other parts of the world as well. During 1984 in Eastern India, epidemics of shigellosis were caused by multidrug resistant *S. dysenteriae* serotype 1; in the same region after 18 years in 2002, the epidemics of shigellosis were found to be caused by *S. flexneri* [24].

In this study, third generation cephalosporins were found to be most sensitive antibiotic (93% of *Shigella* species were sensitive to ceftriaxone) followed by azithromycin. However, 67% of isolates were resistant to amoxicillin, ciprofloxacin and cotrimoxazole. In comparison to *S. flexneri* (83% of isolates were resistant), *S. sonnei* was more sensitive to amoxycillin (all isolates were sensitive). In reverse, 42% of isolates of *S. flexneri* were sensitive to ciprofloxacin and cotrimoxazole, but all isolates of *S. sonnei* were resistant to ciprofloxacin and cotrimoxazole. Similar, kind of result have been shown in a study done by Shah et al. in Nepal [21].

In contrast to the developed world, where *S. sonnei* is common, the occurrence of quinolone resistance is low. For South Asia, *S. flexneri* continues to be the most common serogroup isolated; antibiotic resistance is emerging including ciprofloxacin resistance [24].

In this study, one of the isolates, *S. flexneri* was found to be ESBL-producer which accompanied ciprofloxacin resistance along with third generation cephalosporin. Similarly, ESBL has been reported in *Shigella* species from different parts of the world [10–13].

The ESBL producing *Shigella* was isolated from 59 months old female child. To our knowledge, this is the first report of ESBL producing *Shigella*. The isolate is ESBL-producer with MIC >256 mg/L for cefixime and had positive double disk synergy test and combination disk test both. The isolate was sensitive to cefoxitin, eliminating the possibility of AmpC production. However, the lack of generous molecular laboratory facilities didn’t allow genetic characterization, which can be further quest of further study. The emergence of multidrug-resistant (MDR) strains of *Shigella* is a great concern in South Asia including Nepal [25]. In such a scenario, third-generation cephalosporins are increasingly being used for treating diarrhea where antibiotic is indicated empirically. This, in turn, can lead to the increased prevalence of extended spectrum beta-lactamases (ESBL) producing *Shigella* sp.

## Conclusions

The multi drug resistance is increasing steeply among *Shigella* spp. accompanying third generation cephalosporin resistance as well in some case. So, systemic monitoring of the species and their antimicrobial susceptibility is becoming crucial to guide therapy.

## Abbreviations

AMC: amoxycillin–clavulanic acid
ASM: American Society for Microbiology
ATCC: American type culture collection
BA: blood agar
CAZ: ceftazidime
CD: combination disk
CFU: colony forming units
CLSI: Clinical and Laboratory Standard Institute
CRO: ceftriaxone
D/W: distilled water
ESBL: extended spectrum β-lactamase
Fig: figure
gm: gram
Hrs: hours
K/A: alkaline/acid
LPF: low power field
MA: MacConkey agar
MDR: multi-drug resistant
MHA: Mueller Hinton agar
ml: millilitre
mm: millimetre
No.: number
PMN: polymorphonuclear neutrophils
SIM: sulphide indole motility
TSI: triple sugar iron
TUTH: Tribhuvan University Teaching Hospital
WHO: World Health Organization
µg: microgram
µl: micro litre

## References

[1] World Health Organization. Guidelines for the control of shigellosis including epidemics due to *Shigella dysenteriae* type 1. Geneva, Switzerland. 2005. http://apps.who.int/iris/bitstream/10665/43252/1/924159330X.pdf. Accessed 5 Nov 2014.

[2] Lima AM, Lima NL, Pinho CNM, Barros EA, Teixeira MJ, Martins MC, Guerrant RL (1995). High frequency of strains multiply resistant to ampicillin, trimethoprim–sulfamethoxazole, streptomycin, chloramphenicol, and tetracycline isolated from patients with shigellosis in Northeastern Brazil during the period 1988 to 1993. Antimicrob Agents Chemother.

[3] Niyogi SK (2005). Shigellosis. J Microbiol.

[4] Von Seidlein L, Kim DR, Ali M, Lee H, Wang X, Thiem VD, do Canh G, Chaicumpa W, Agtini MD, Hossain A, Bhutta ZA, Mason C, Sethabutr O, Talukder K, Nair GB, Deen JL, Kotloff K, Clemens JA (2006). Multicentre study of Shigella diarrhoea in six Asian countries: disease burden, clinical manifestations, and microbiology. PLoS Med.

[5] Tajbakhsh M, García Migura L, Rahbar M, Svendsen CA, Mohammadzadeh M, Zali MR, Aarestrup FM, Hendriksen RS (2012). Antimicrobial resistant *Shigella* infections from Iran: an overlooked problem?. J Antimicrob Chemother.

[6] Zhang W, Luo Y, Li J, Lin L, Ma Y, Hu C, Jin S, Ran L, Cui S (2011). Wide dissemination of multidrug-resistant *Shigella* isolates in China. J Antimicrob Chemother.

[7] Fortineau N, Naas T, Gaillot O, Nordmann P (2001). SHV-type extended-spectrum β-lactamase in a *Shigella flexneri* clinical isolate. J Antimicrob Chemother.

[8] Pai H, Choi EH, Lee HJ, Hong JY, Jacoby GA (2001). Identification of CTX-M-14 extended-spectrum β-lactamase in clinical isolates of *Shigella sonnei, Escherichia coli* and *Klebsiella pneumonia* in Korea. J Clin Microbiol.

[9] Radice M, González C, Power P, Vidal MC, Gutkind G (2001). Third-generation cephalosporin resistance in *Shigella sonnei* Argentina. Emerg Infect Dis..

[10] Nguyen NT, Ha V, Tran NV, Stabler R, Pham DT, Le TM, van Doorn HR, Cerdeño-Tárraga A, Thomson N, Campbell J, Nguyen VM, Tran TT, Pham MV, Cao TT, Wren B, Farrar J, Baker S (2010). The sudden dominance of blaCTX-M harbouring plasmids in *Shigella* spp. circulating in Southern Vietnam. PLoS Negl Trop Dis..

[11] Acikgoz ZC, Gulay Z, Bicmen M, Gocer S, Gamberzade S (2003). CTX-M-3 extended-spectrum β-lactamase in a *Shigella sonnei* clinical isolate: first report from Turkey. Scand J Infect Dis.

[12] Bhattacharya D, Bhattacharjee H, Ramanathan T, Sudharma SD, Singhania M, Sugunan AP, Roy S (2011). Third generation cephalosporin resistance in clinical isolate of *Shigella sonnei* in Andaman & Nicobar Islands India. J Infect Dev Ctries.

[13] Chander A, Shrestha CD (2013). Prevalence of extended spectrum beta lactamase producing *Escherichia coli* and *Klebsiella pneumoniae* urinary isolates in a tertiary care hospital in Kathmandu, Nepal. BMC Res Notes.

[14] Shrestha S, Amatya R, Dutta R (2011). Prevalence of extended spectrum beta lactamase (ESBL) production in gram negative isolates from pyogenic infection in tertiary care hospital of Eastern Nepal. Nepal Med Coll J..

[15] Mishra KS, Kattel H, Pokhrel BM (2015). High prevalence of extended-spectrum-beta-lactamase- producing respiratory bacterial pathogens in a Nepalese University Hospital: a vexatious problem. ACCLM.

[16] Paterson DL, Bonomo RA (2005). Extended-spectrum beta-lactamase: a clinical update. Clin Microbiol Rev.

[17] Rupp ME, Fey PD (2003). Extended spectrum β-lactamase (ESBL)—producing enterobacteriaceae: considerations for diagnosis, prevention and drug treatment. Drug..

[18] Henry DI (2004). Clinical microbiology procedures handbook.

[19] Sharon ME, Elizabeth EB, Janet FH (2008). Options for treating resistant *Shigella* species infections in children. J Pediatr Pharmacol Ther..

[20] Clinical and Laboratory Standards Insitute (2009). Performance standards for antimicrobial susceptebility testing: nineteenth informational supplement M100-S19.

[21] Shah BK, Sharma S, Shakya G, Upadhyay BP (2012). Multidrug resistant *Vibrio cholera*, *Salmonella* and *Shigella* from Nepalgunj cholera outbreak and different hospitals of Nepal. Nepal J Biosci.

[22] Bhattacharya S, Khanal B, Bhattarai NR, Das ML (2005). Prevalence of *Shigella* species and their antimicrobial resistance pattern in Eastern Nepal. J Health Popul Nutr.

[23] Kansakar P, Malla S, Ghimire GR (2007). *Shigella* isolates of Nepal: changes in incidence of *Shigella* subgroups and trends of antimicrobial susceptibility pattern. KUMJ..

[24] Taneja N (2007). Changing epidemiology of shigellosis and emergence of ciprofloxacin-resistant *Shigella* in India. J Clin Microbiol..

[25] Rahman M, Shoma S, Rashid H, Siddique AK, Nair GB, Sack DA (2004). Extended spectrum beta-lactamase mediated third generation cephalosporin resistance in *Shigella* isolates in Bangladesh. J Antimicrob Chemother.

